# Traumatic lumbar disc herniation: A systemic case review and meta-analysis

**DOI:** 10.1016/j.bas.2023.102350

**Published:** 2023-08-19

**Authors:** J. Li, L. Gössel, B. Kunze, O. Kessler, Y. Alharbi, O. Gärtner, V.A. Mihalca, S. Krebs, M. Dreimann

**Affiliations:** a Spine Center for Neuroorthopaedics, Spinal Cord Injuries, and Scoliosis, RKH Orthopedic Clinic Markgröningen, Markgröningen, Germany

**Keywords:** Traumatic lumbar disc herniation, Extruded disc herniation, Spine, Trauma

## Abstract

**Introduction:**

Traumatic lumbar disc herniation (TLDH) without fracture in the *in-situ* motion segment is a rare occurrence compared with degenerative herniation.

**Research question:**

This study provides a systematic discussion of various aspects related to the diagnosis of TLDH.

**Material and methods:**

This review includes 12 cases of TLDH with MR-images since 2009 published in the PubMed and one adjunct illustration. The cases were categorized into two groups for a comprehensive analysis, TLDH with or without *in-situ* segment fracture. Additionally, we reported a case of a 43-year-old female patient with a recent stenosing TLDH at L5/S1, accompanied by a large sequestration (disc herniation stage-4, and Michigan State University Classification: MSU 3-AB) and an endplate compression fracture at L2 (AO A1).

**Results:**

Isolated traumatic lumbar disc herniation is possible, but it is required exclude cases with fractures in the *in-situ* motion segment.

**Discussion and conclusion:**

Trauma with related injury mechanisms is the highest priority for the diagnosis of TLDH. Low-grade disc degeneration without significant instability could be accepted for diagnosing TLDH. A TLDH on MR images might show a slightly lower T2-signal compared to the CSF and a homogeneous T1-signal like the spinal cord, as well as a similar STIR-signal of the sequestration and CSF. If necessary, a histological examination could be performed to evaluate the degenerative changes in the injured disc, especially to assist the evaluation due to legal reasons.

## Highlights

• We reviewed 12 reports of TLDH with MR-images since 2009. A comparative analysis of the following aspects in each case, including age, gender, trauma mechanism, damaged segment, vertebral fracture, neural deficits, surgeries, imaging changes of disc degeneration, initial misdiagnosis, and final diagnosis was performed.
• Isolated TLDH is possible, but it is required exclude fractures in the in-situ motion segment. Trauma is the highest priority for the diagnosis of TLDH.
• Radiologically, mild disc degeneration without instability should be accepted for diagnosing TLDH. The characteristics of a TLDH on MR images might show a slightly lower T2-signal than the CSF and a homogeneous T1-signal like the spinal cord, as well as a similar STIR-signal of the sequestration and CSF. If necessary, a histological examination could be performed to evaluate the degeneration.

## 1 Introduction

Lumbar disc herniation (LDH) is usually considered as a non-traumatic pathological condition (Benzakour et al., 2019), resulting from either in young patients with genetic disorders (Kalb et al., 2012; Jiang et al., 2023) or in older patients with degenerative changes (Kos et al., 2019). Trauma is a rare cause of LDH, and a diagnosis of purely traumatic lumbar disc herniation (TLDH) is also difficult to confirm. However, cases of TLDH have been reported since the 1850s, and some cases are still occasionally reported and discussed today. The main concerns are focused on a reliable diagnosis based on a causal chain: if there is no high-grade disc degeneration, then a diagnosis of TLDH should be considered following an adequate trauma.

TLDH can cause symptoms like degenerative LDH, such as low back pain, sciatica, paresthesia, and muscle weakness in the legs. In some extreme cases, cauda equina syndrome with bladder and sphincter dysfunction may occur. The locations of the extruded disc mass due to trauma can vary from epidural to intradural. The disc mass often appears as a complete block with a larger size. For intradural or transdural located disc complexes, fine neurosurgical techniques would be necessary to explore the intradural space and probably repair the dura. A vertebral fracture at the *in-situ* motion segment should be excluded for the diagnosis of TLDH.

## 2 The review of TLDH cases

### 2.1 Methods

We searched for relevant case reports on PubMed by using “traumatic lumbar disc herniation” as the keyword. The earliest report on traumatic disc injury can be traced back to 1929 (Dandy, 1929), and since the mid-20th century, discussions on traumatic disc herniation have continued (Frowein and Terhaag, 1977; Voigt and Marquardt, 1981; Laun, 1983; Ando and Mimatsu, 1993). We reviewed 12 reports of TLDH with MR-images since 2009 (Table 1, Table 2). A comparative analysis of the following aspects in each case, including age, gender, trauma mechanism, damaged disc segment, concomitant vertebral fracture, neural deficits, surgical treatment, imaging changes of intervertebral disc degeneration before trauma, initial misdiagnosis, and final diagnosis was performed. The cases were categorized into two groups for a comprehensive analysis. Group A: Cases of misdiagnosis of TLDH with fracture at the in-situ vertebral motion segment and, Group B: Cases of true TLDH without fracture at the *in-situ* vertebral motion segment.

**Table 1:** 6 cases of spinal fracture with intervertebral disc injury and spinal stenosis, originally reported as traumatic lumbar disc herniation. The average age is 30.50 ± 13.03 Mean ± SD.

Author	Year	Age	Sex	Inj. Disc	Fraktur	Trauma	Neurology	Operation	Signs of Degeneration	Primary misdiagnosis	End Diagnose
Lee (Lee et al., 2020)	2020	32	M	L2/3	L2 (AO A1)	Fall	Paraparesis and paresthesia	Spinal Fusion with Decomp.	L4/5 Pfirrmann III with disc height reduction	SEH	L2 fracture (AO A1) combined with a dural defect and damaged nerve roots by a transdural sequester
Kim (Kim et al., 2019)	2019	39	M	L3/4	T12-L2 (AO A1) L4 (AO A4)	Hit on Back	Paraparesis and paresthesia	Spinal Fusion with Decomp.	L2/3, 4/5 Pfirrmann III-IV with disc height reduction	SEH	T12-L2 fractures (AO A1) and L4 fracture (AO A4) with ventral epidural sequester
Kadam (Kadam et al., 2017)	2017	15	M	L2/3	L2 apophyseal fracture	Fall	Paraparesis	Decomp.	L1/2, 2/3 und L5/S1 Pfirrmann II-III with disc height reduction	No	Apophyseal fracture of the L2 inferior endplate combined with disc protrusion L2/3 and spinal stenosis
Pourabbas (Pourabbas et al., 2016)	2016	22	M	L1/2 L5/S1	L1/2 (AO B2) L5/S1 (AO C)	Fall	Paraparesis	Spinal Fusion with Decomp.	L1/2 und 4/5 Pfirrmann II, L5/S1 Pfirrmann III with disc height reduction	No	L1/2 fracture (AO B2) with annular tear L5 fracture (AO C) with traumatic retrolisthesis and sequester
Song (Song and Yang, 2011)	2011	24	M	T12/L1	T12 (AO A4)	Fall	Initially No. In 4 days: Paraplegia, paresthesia, Babinski sign, and Conus medullaris syndromes	Spinal Fusion with Decomp.	M. Scheuermann, Schmorl's node T12 with disc height reduction	PREMDH	T12 fracture (AO A4) with PREMDH combined with severe conus medullaris syndromes by large Sequester
Jang (Jang et al., 2010)	2010	51	M	L1/2	L1 (AO A1)	Traffic accident	paraparesis and hypesthesia	Decomp.	L1/2 Pfirrmann III with disc height reduction	SEH, Neoplasm	L1 fracture (AO A1) with intradural Sequester

SEH: Spinal epidural hematoma. PREMDH: posterior retroextramarginal disc hernia. Decomp.: Operative Decompression. AO: AO Classification of fractures.

**Table 2:** 7 cases of traumatic lumbar disc herniation without *in-situ* spinal fracture. The average age is 37.14 ± 11.22 Mean ± SD.

Author	Year	Age	Sex	Inj. Disc	Fraktur	Trauma	Neurology	Operation	Degeneration	Primary misdiagnosis	End Diagnose
Our case	2023	42	W	L5/S1	L2 (AO A1)	Fall	Short-term paresis, fully recovered without surgery	No	L5/S1 Pfirrmann II	No	L2 (AO A1) fracture. L5/S1 TLDH with ventral epidural sequester
Basile (Basile et al., 2020)	2020	27	M	L4/5	No	Fall	Paraparesis and paresthesia	Decomp.	L4/5, L5/S1 Pfirrmann III with disc height reduction	SEH	L4/5 TLDH with bilateral ventral epidural sequester
Ahmed (Ahmed et al., 2019)	2019	35	M	L4/5	No	Horse Kick Abdomen	No	No	L4/5 Pfirrmann III with disc height reduction	No	L4/5 TLDH
Jain (Jain et al., 2018)	2018	50	M	L2/3	No	Hit on Back	No	Decomp.	L4/5 Pfirrmann III with disc height reduction	SEH	L2/3 TLDH with Ventral epidural sequester
Lee (Lee and Kwon, 2013)	2013	52	M	L1/2	No	Fall	Paresthesia	Decomp.	L1/2 calcification with bony spur	SDH	L1/2 TLDH with ventral dura defect and intradural sequester
									L1/2 and 4/5 Pfirrmann III with disc height reduction		
Song (Song et al., 2012)	2012	23	M	L4/5	No	Traffic accident	Paraparesis and paresthesia	Decomp.	L4/5 und L5/S1 Pfirrmann II	SEH	L4/5 TLDH with ventral epidural sequester
González (González-Bonet and Mollá-Torró, 2009)	2009	31	W	L5/S1	No	Traffic accident	initially No In 2 days: cauda equina syndrome	Decomp.	L5/S1 Pfirrmann IV, posterolateral lumbar disc herniation before accident, Osteochondrose	No	L5/S1 TLDH with ventrodorsal epidural Sequester and cauda equina syndrome

SEH: Spinal epidural hematoma. SDH: Spinal subdural hematoma. Decomp.: Operative Decompression. AO: AO Classification of fractures.

### 2.2 Group A: cases of misdiagnosis of TLDH with fracture at the in-situ vertebral motion segment

After conducting a detailed analysis of 13 cases, we discovered an interesting feature. Although 6 cases (Table 1) reported TLDH with spinal stenosis, all had fresh vertebral fractures of the *in-situ* vertebral motion segment. This means that the diagnosis for these 6 cases should be spinal fracture with intervertebral disc injury and spinal stenosis rather than TLDH with spinal stenosis.

**Fig. 1:**
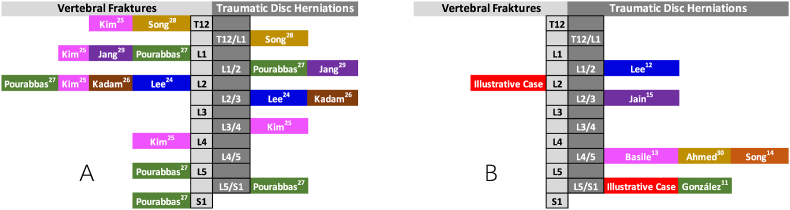
The distribution of TLDH and fractures. A: The reported misdiagnosed TLDH-cases with fractures in *in-situ* motion segment; B: The reported true TLDH-cases without fractures in *in-situ* motion segment.

**Fig. 2:**
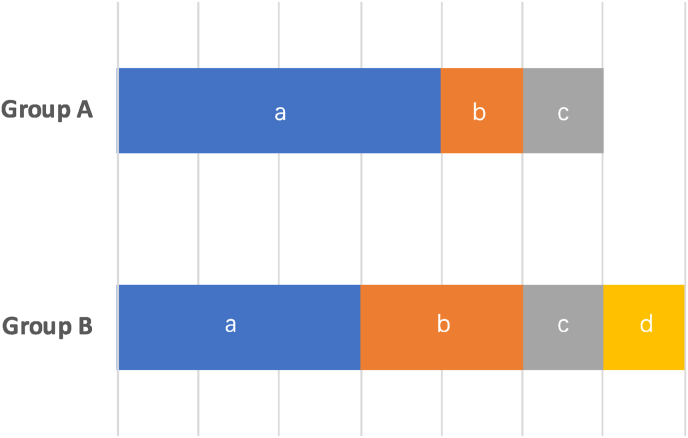
Trauma in Group A and B. (a): Fall; (b): Traffic accident; (c): Hit on back; (d): Hit on abdomen.

The patients' ages ranged from 15 to 51 years old with an average age of 30.50 ± 13.03 Mean ± SD, and all 6 patients were male. Disc injuries occurred at all lumbar disc levels, from T12/L1 to L5/S1. The most common location for fractures in all 6 patients was the proximal lumbar spine (Fig. 1 A), which is consistent with the most frequent region for spinal fractures, the thoracolumbar region (Mokhtarzadeh et al., 2021). The type of fracture according to the AO classification included A1, A4, B2, and C. Falls was the most common cause of spinal injuries, followed by traffic accidents and being hit on the back (Fig. 2).

The patients exhibited varying degrees of neurological deficits, including paraparesis, paresthesia, and loss of rectal tone and perianal sense as a cauda eqina syndrome. All patients underwent decompression surgery, with most of them requiring spinal fusion because of the instability caused by the fractures. Before trauma, the intervertebral discs had mostly reached Pfirrmann II-III with a slight reduction in height, except for one patient with M. Scheuermann and Schmorl's node. The primary misdiagnosis often a spinal epidural hematoma, with one case of posterior retroextramarginal disc herniation and another of a neoplasm. Two cases with dural defects were reported. One had intradural sequestration (Jang et al., 2010) and the other had transdural sequestration (Lee et al., 2020). The Transdural sequestration comprises two lesions: an intradural mass-like lesion on the ventral side of the spinal cord and an epidural mass-like lesion on the dorsal side, which raised suspicion of connectivity between them.

### 2.3 Group B: cases of true TLDH without fracture at the *in-situ* vertebral motion segment

In contrast, the seven cases of TLDH without *in-situ* fractures exhibited vastly different characteristics. The patients' ages ranged from 23 to 52 years old with an average age of 37.14 ± 11.22 Mean ± SD, with most males (5 out of 7 patients). Of the seven cases, three patients had falls injuries, two were involved in traffic accidents, and the last two had suffered blunt abdominal and back injuries, respectively. Fall injuries no longer feature prominently in the trauma mechanisms of fracture-free patients (Fig. 2). Unlike the cases with fractures, the injured lumbar discs without *in-situ* spinal fractures were mainly located in the lower lumbar spine, with L4/5 and L5/S1 being the most affected levels (Fig. 1 B).

Regarding neurological deficits, most of the patients experienced various short-term neurological issues, including paresis, paraparesis, and paresthesia. Only one patient exhibited cauda equina syndrome. Two of seven patients fully recovered without surgery, while the others required only decompression surgery. None of the patients underwent spinal fusion. In terms of lumbar disc degeneration, four patients showed signs of disc degeneration in Pfirrmann grade III with mild disc height reduction, and two patients had disc degeneration in Pfirrmann grade II. One unique patient had not only Pfirrmann grade IV disc degeneration with reduced disc height but also a pre-traumatically diagnosed herniated disc in the injured L5/S1 disc (González-Bonet and Mollá-Torró, 2009). The herniated disc at L5/S1 worsened after the trauma, which caused the lumbar disc complex to extrude into the spinal canal. This led to spinal canal stenosis and compression of the cauda equina, resulting in cauda equina syndrome. One of the four patients with Pfirrmann grade III disc degeneration at L1/2 underwent surgical decompression after two weeks of conservative treatment for suspected subdural hematoma and persistent paresthesia. During the operation, a ventral dural defect caused by a dorsal bony spur at the level of L1/2 with intradural disc complex was identified (Lee and Kwon, 2013).

## 3 Illustrative case

**Fig. 3:**
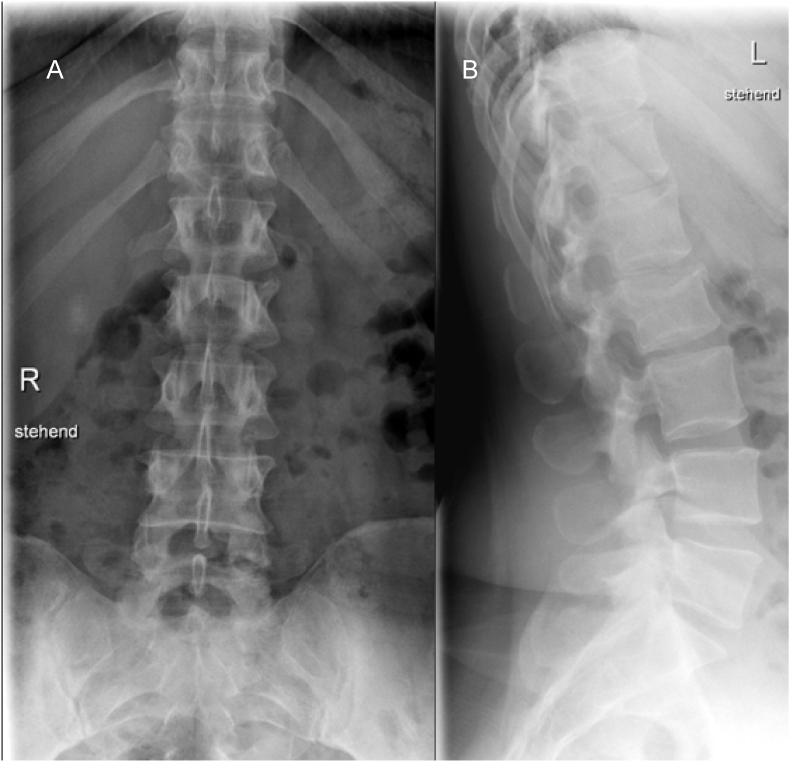
X-ray of the lumbar spine (AP and lateral) after the trauma. An endplate compression fracture without the posterior edge involvement is shown in the lateral image.

A 43-year-old female patient was transferred to the spine center due to left foot drop paralysis and back pain with radiating left leg pain, corresponding to the L4 dermatome, from a local hospital. Two days earlier, the patient was sitting on her horse for a walk. Her horse became restless without warning and attempted to throw her off the horse by jumping. After being thrown from the horse, she first touched the ground with her hips and then lay on her back. This accident was an adequate trauma to induce TLDH. She was admitted to the local hospital by ambulance. Analgesic therapy with ibuprofen and tapentadol for an endplate compression fracture at L2 (AO A1) confirmed by X-ray (Fig. 3) was administered at the local hospital. Due to the presence of left foot drop paralysis two days after the accident (grade 3/5 strength) and persistent numbness of the left lateral edge of the foot and lateral posterior thigh (Dermatome S1 left), the patient was transferred to the spine center for further treatment. The patient did not report any bladder, bowel or pudendal dysfunctions. The left foot drop paralysis was fully restored in the meantime, but hypesthesia along the dermatome S1 on the left side persisted. Symptoms with pain improved significantly after further conservative therapy with metamizole and tapentadol, and physiotherapy and mobilization. The patient was discharged from department after seven days of inpatient pain therapy.

**Fig. 4:**
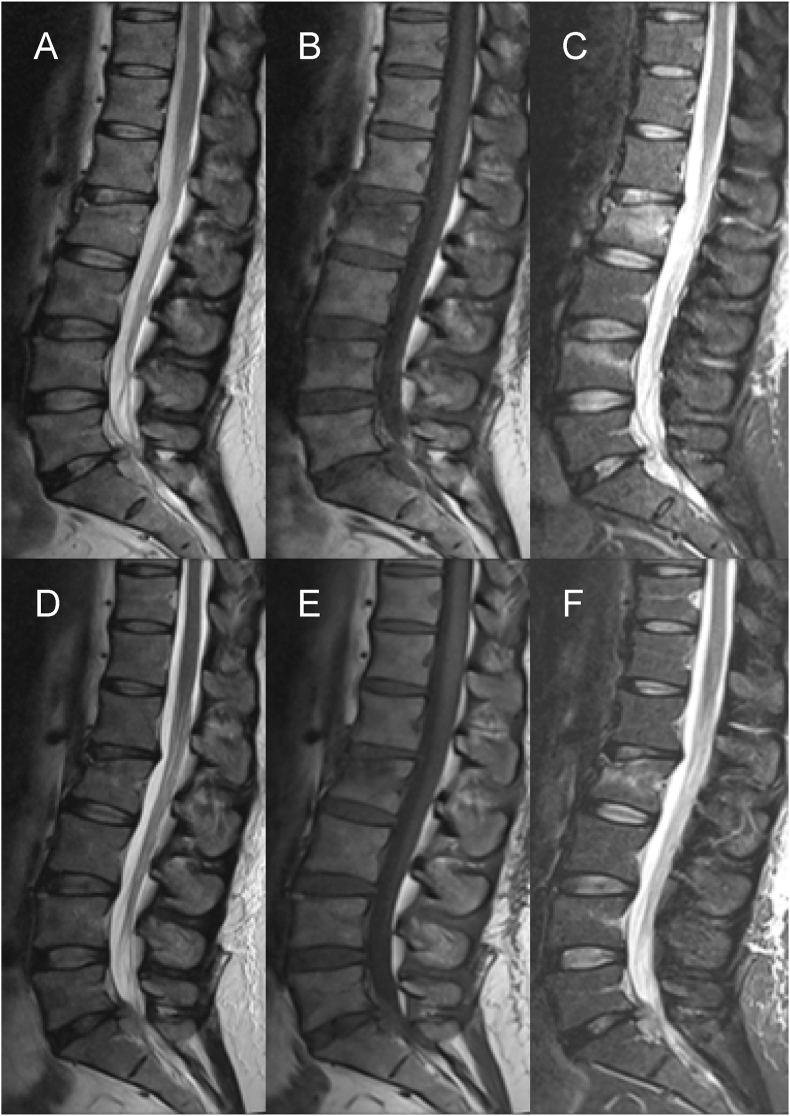
(MRI-after the trauma: A-T2 weight, B-T1 weight, and C-stir) The remaining disc tissue in the L5/S1 disc space and the giant sequester showed slightly weakened hyperintensity on T2 signal compared to the cerebrospinal fluid, as well as homogeneous hypointensity on T1 signal like the spinal cord. The STIR-intensities of sequestration and CSF were almost the same. The MRI findings at the follow-up (D-T2 weight, E-T1 weight, and F-stir) showed a further decrease in T2 signal intensity (D) compared to the MRI obtained one month prior (A). The difference in T2-intensity (D) and STIR-intensity (F) between the sequestration and liquor signal was more significant (D). The volume of intraspinal sequestration slightly decreased.

**Fig. 5:**
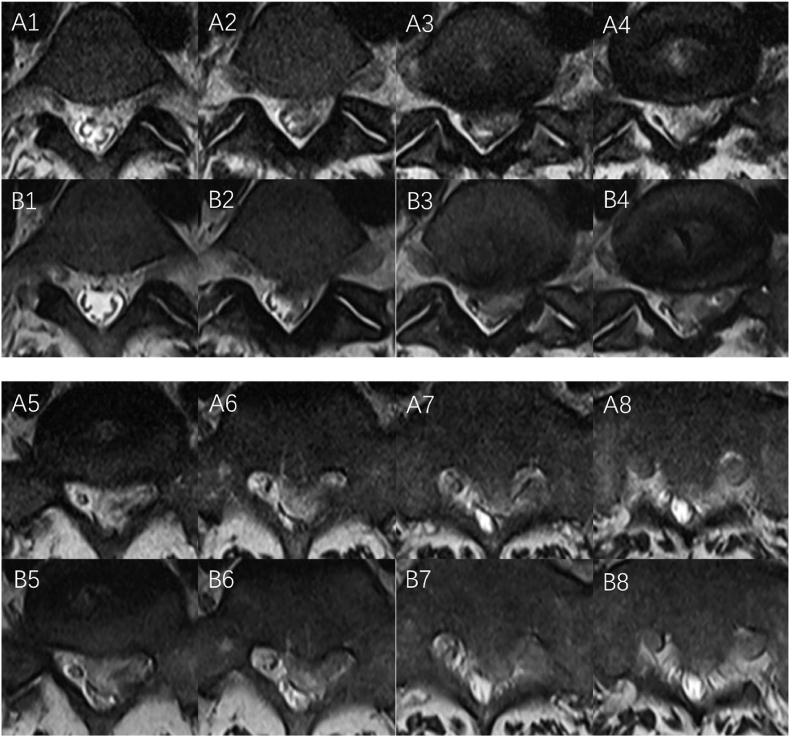
(MRI, Axial images of motion segment L5/S1 (T2-Signal): A1-A8: Images after the injury, from cranial to caudal: giant epidural ventral sequestration in the spinal canal. The sequestration in image A4-A6 showed a slightly lower T2-signal in comparison to CSF. B1–B8: Images by follow-up one month after the injury, from cranial to caudal The images showed a further decrease in T2-signal compared to the MRI after the injury. The decreased T2-signal of the sequestration in comparison with CSF was more significant (A3/B3, A4/B4, A5/B5, A6/B6). The compression on Dura was slightly reduced (A4/B4, A5/B5, A6/B6, A7/B7, A8/B8).

The patient returned one month after the accident for clinical and radiological follow-up. She reported a significant improvement in pain and currently experiences hardly any pain in the lumbar spine region. She regularly wore a three-point brace when standing and walking and only took analgesics as needed. The patient reported persistent tingling paresthesia in dermatome S1 left but did not present with any new lower extremity paralysis or bladder/rectal dysfunctions. The MRI findings at the follow-up were compared to those obtained after the injury, which showed a further decrease in T2-intensity compared to the MRI obtained one month prior (Fig. 4, Fig. 5). The difference in T2-intensity and STIR-signal between the sequestration and liquor signals was more significant. The volume of intraspinal sequestration slightly decreased.

MRI of the lumbar spine was performed (Fig. 4, Fig. 5) in the spine center, which revealed a fresh stenosing herniated disc L5/S1 with sequestration through the ruptured posterior longitudinal ligament (Disc Herniation Stage 4, and Michigan State University Classification: MSU 3-AB) and caudal migration in the L5/S1 segment (Fig. 4, Fig. 5). Sequestration caused moderate narrowing of the spinal canal and affected the adjacent cauda fibers, as well as moderate narrowing of the neuroforamina L5/S1 on both sides. The remaining disc tissue in the L5/S1 disc space and the giant sequestration showed a slightly weakened inhomogeneous T2 signal compared to the liquor signal, as well as a homogeneous T1 signal similar to the spinal cord. The STIR intensities of sequestration and cerebrospinal fluid (CSF) were almost the same. Additionally, our MRI revealed a fresh endplate compression fracture of the L2 vertebra with a cortical contour disruption without the posterior edge involvement (AO A1). A very discreet bone marrow edema in the L4 cranial/left without vertebral height reduction or posterior edge displacement was detectable. No intraspinal hematoma was visible (Fig. 4).

The patient denied any symptoms related to lumbar degenerative changes before the trauma, therefore, there were no MRI images of the lumbar spine before the trauma for comparison.

## 4 Discussion

### 4.1 Clinically

TLDH is a pathological condition in which an intervertebral disc is injured due to trauma, causing the disc complex to extrude into the spinal canal and leading to spinal stenosis. The extruded intervertebral disc complex with rupture of the posterior longitudinal ligament comprises all disc materials, including the nucleus pulposus, parts of the annulus fibrosus, and probably cartilaginous endplates. The location of the extruded disc materials in the spinal canal can vary greatly, as they can be located in the ventral or post-epidural space^13 11 14 15^, or could be found trans- or intradural with the presence of an injury on dura (Lee and Kwon, 2013). Neurological symptoms in the lower extremities are not dependent on the size or location of the prolapsed intervertebral disc tissue. These neurological symptoms can range from leg pain and paresthesia to paralysis and loss of the bladder or bowel control. In some cases, patients may not have any neurological symptoms initially, but these symptoms can rapidly deteriorate over a short period of time, ultimately resulting in sphincter dysfunction (González-Bonet and Mollá-Torró, 2009). In terms of time, the extruded intervertebral disc complex can lead to neurological deficits even up to 6 weeks after the initial accident (Lee and Kwon, 2013). Our case did not exhibit any lower extremity neurological deficits during initial inpatient pain therapy or at the one-month outpatient follow-up, except for slight hypesthesia in dermatome S1 left.

#### 4.1.1 MR-imaging for TLDH

The most frequent misdiagnosis of TLDH in MRI is hematoma, such as spinal epidural hematoma (SEH) or spinal subdural hematoma (SDH). Four out of seven true-TLDH patients were initially misdiagnosed with intraspinal canal hematoma. MRI images of acute spinal hematoma were reported as isointense or hypointense on T1-signal, while subacute and chronic hemorrhages may show high T1-signal and heterogeneity or mixed hyper/hypointensity on T2-signal (Basile et al., 2020). According to Song et al., the indication of a TLDH is low T2-signal and high T1-signal. Unlike intraspinal hematomas, traumatic herniated discs usually shrink over time. However, this indication corresponds to a severely degenerated intervertebral disc with instability, such as Pfirrmann grade III-V, which excludes the diagnosis of TLDH.

In the illustrative case, the remaining disc tissue in the L5/S1 disc space and the giant sequestration showed only a slightly lower T2-signal compared to the CSF, as well as a homogeneous T1 signal like the spinal cord. The STIR signals of sequestration and CSF were almost the same. These MRI signals are consistent with the features of a Pfirrmann grade II intervertebral disc, which indicates that no degenerative instabilities were involved.

#### 4.1.2 Histological examination for TLDH

A histopathological examination of the intervertebral disc tissue collected during the operation and the use of a histologic degeneration scoring (HDS) (Weiler et al., 2011) to evaluate the degeneration in the damaged disc is recommended. This can effectively reduce the diagnostic uncertainty of TLDH. HDS is a scoring system that evaluates different features in the disc tissue, including chondrocyte proliferation, granular changes, structural alterations that involve tears and clefts, and mucous degeneration, which is characterized by dark blue staining areas surrounding chondrocyte clusters. Additionally, an analysis of gene transcription data was reported to distinguish whether degeneration is the primary cause of TLDH (Dudli et al., 2014).

#### 4.1.3 Suggestions for the diagnosis of TLDH

In addition to trauma as the main cause of disc herniation, several prerequisites should be considered. First, a spinal fracture (AO A-C) at the *in-situ* motion segment of TLDH should be excluded. Second, moderate or severe degenerative changes and degenerative spinal instability with progressive loss of disc space height in the lumbar motion segment of TLDH should be excluded. For instance, disc degeneration in Pfirrmann Grade III-V or modified Pfirrmann Grade 5–8, or osteochondrosis in Modic type 2 and 3 (Zhang et al., 2008). Third, a pronounced degenerative spondylophyte in the motion segment of TLDH should also be excluded because of the possibility of puncturing the dura and causing trans- or intradural sequestration (Lee and Kwon, 2013). Fourth, signs of severe lumbar instability also need to be excluded, such as severe spondylolisthesis, spondylolysis, and severe scoliotic changes. Last but not the least, if necessary, the intervertebral disc tissue removed during surgical decompression can be sent for histological examination to determine the extent of its degenerative changes. This may be particularly relevant in cases where a TLDH is claimed to be a legal problem.

### 4.2 Economically

In fact, the TLDH debate is not centered around its clinical diagnosis and treatments, but rather on defining it as a work-related injury. Controversies regarding TLDH and their evaluation in social accident insurance have been present since the 1950s (Lob and Unghanns, 1959). In 1990, Lemke et al. extensively discussed the essential aspects that must be considered when determining work-related TLDH (Lemke and Manthei, 1990). In an expert assessment, Schwarze et al. proposed a scheme for evaluating the TLDH (Schwarze et al., 2017). A classification of traumatic disc herniation based on image morphology by Sander et al. was involved in this expert recommendation (Sander et al., 2013). This classification, however, primarily describes edema in the disc, disc rupture with bleeding and protrusion into the vertebral body, and tears in the fibrous ring or herniation in the endplate. It does not include the terms “disc herniation” or “sequestration”, nor does it address spinal canal stenosis resulting from these herniations. For patients with TLDH who have undergone surgical treatment, it is also necessary to consider the possibility of developing chronic pain during follow-up.

## 5 Conclusion

Trauma with related injury mechanisms is the highest priority for the diagnosis of TLDH. A fracture in the *in-situ* motion segment of TLDH should be ruled out. Radiologically, mild disc degeneration without spinal instability should be accepted for diagnosing TLDH. The characteristics of a TLDH with mild disc degeneration on MR images might show a slightly lower T2-signal compared to the CSF and a homogeneous T1-signal like the spinal cord, as well as a similar STIR-signal of the sequestration and CSF. If necessary, a histological examination could be performed to distinguish whether degeneration is the primary cause of TLDH, especially to assist the evaluation of work-related TLDH.

## Declaration of generative AI and AI-assisted technologies in the writing process

During the preparation of this work the author (J. Li) used [Trinka Grammar] in order to [check the English grammar]. After using this tool, the author (J. Li) reviewed and edited the content as needed and takes full responsibility for the content of the publication.

## Declaration of competing interest

The authors declare that they have no known competing financial interests or personal relationships that could have appeared to influence the work reported in this paper.
